# Nuclear pyruvate kinase M2 complex serves as a transcriptional coactivator of arylhydrocarbon receptor

**DOI:** 10.1093/nar/gkv967

**Published:** 2015-09-23

**Authors:** Shun Matsuda, Jun Adachi, Masaru Ihara, Nobuhiro Tanuma, Hiroshi Shima, Akira Kakizuka, Masae Ikura, Tsuyoshi Ikura, Tomonari Matsuda

**Affiliations:** 1 Research Center for Environmental Quality Management, Kyoto University, Shiga 520-0811, Japan; 2 Laboratory of Proteome Research, National Institute of Biomedical Innovation, Osaka 567-0085, Japan; 3 Division of Cancer Chemotherapy, Miyagi Cancer Center Research Institute, Miyagi 981-1293, Japan; 4 Laboratory of Functional Biology, Kyoto University Graduate School of Biostudies, Kyoto 606-8501, Japan; 5 Laboratory of Chromatin Regulatory Network, Department of Mutagenesis, Radiation Biology Center, Kyoto University, Kyoto 606-8501, Japan

## Abstract

Pyruvate kinase M2 (PKM2) and pyruvate dehydrogenase complex (PDC) regulate production of acetyl-CoA, which functions as an acetyl donor in diverse enzymatic reactions, including histone acetylation. However, the mechanism by which the acetyl-CoA required for histone acetylation is ensured in a gene context-dependent manner is not clear. Here we show that PKM2, the E2 subunit of PDC and histone acetyltransferase p300 constitute a complex on chromatin with arylhydrocarbon receptor (AhR), a transcription factor associated with xenobiotic metabolism. All of these factors are recruited to the enhancer of AhR-target genes, in an AhR-dependent manner. PKM2 contributes to enhancement of transcription of *cytochrome P450 1A1* (*CYP1A1*), an AhR-target gene, acetylation at lysine 9 of histone H3 at the *CYP1A1* enhancer. Site-directed mutagenesis of PKM2 indicates that this enhancement of histone acetylation requires the pyruvate kinase activity of the enzyme. Furthermore, we reveal that PDC activity is present in nuclei. Based on these findings, we propose a local acetyl-CoA production system in which PKM2 and PDC locally supply acetyl-CoA to p300 from abundant PEP for histone acetylation at the gene enhancer, and our data suggest that PKM2 sensitizes AhR-mediated detoxification in actively proliferating cells such as cancer and fetal cells.

## INTRODUCTION

Acetyl-CoA is a central metabolite straddling diverse metabolic pathways, including glycolysis, the citric acid cycle, fatty acid oxidation, lipid biosynthesis, ketogenesis and amino acid metabolism. It also participates in signal transduction as an acetyl donor for protein acetylation. In the nucleus, histone acetylation drives chromatin structural changes to facilitate DNA transcription, replication and repair (reviewed in (1)). While it is well known that acetyl-CoA production systems exist in mitochondria and the cytoplasm, recent reports have revealed that the nucleus also possesses such systems (2–4). However, the mechanism by which acetyl-CoA is made available for histone acetylation in a gene-specific context is still not clear.

Pyruvate kinase and pyruvate dehydrogenase complex (PDC) regulate a large portion of acetyl-CoA production from glucose intermediates. Pyruvate kinase is a rate-limiting glycolytic enzyme which transfers the phosphate group of phosphoenolpyruvate (PEP) to adenosine diphosphate (ADP) to produce pyruvate and adenosine triphosphate (ATP). PDC is a mitochondrial protein complex which irreversibly decarboxylates pyruvate to produce acetyl-CoA, thus connecting glycolysis and the citric acid cycle. However, recent reports indicate there is significant crosstalk between these enzymes and nuclear transcription. Among the four isoforms of pyruvate kinase, the proliferating cell-specific M2 isoform (PKM2) functions as a co-activator for several transcription factors involved in regulation of cell proliferation and maintenance of an undifferentiated state (5–8). It has also been reported that PKM2 enhances transcriptional activity via its protein kinase activity, for example through phosphorylation of Tyr705 of STAT3 (5) or Thr11 of histone H3 (9). PDC-E2, one of three PDC subunits, interacts with STAT5 in the nucleus to enhance STAT5 transcriptional activity (10). However, whether or how these enzymes orchestrate transcriptional activation is still unknown.

Here, we demonstrate that PKM2, PDC-E2 and histone acetyltransferase (HAT) p300 constitute a complex in the nucleus with arylhydrocarbon receptor (AhR), a ligand-activated transcription factor involved in activating genes related to xenobiotic metabolism such as *cytochrome P450 1A1* (*CYP1A1*) (11). All these factors (PKM2, PDC-E2 and p300) are recruited to the *CYP1A1* enhancer in an AhR-dependent manner. Furthermore, we show that PDC activity is present in the nucleus and that the pyruvate kinase activity of PKM2 enhances histone H3K9 acetylation at the enhancer. Based on our findings, we suggest that PKM2 sensitizes AhR-mediated detoxification in cancer cells and fetal cells to ensure proliferation, and propose a ‘local acetyl-CoA production’ model for transcriptional activation.

## MATERIALS AND METHODS

### Cell culture

Human cervical cancer HeLa S3 cells were cultured in Dulbecco's modified Eagle's medium (DMEM) supplemented with 10% fetal calf serum. Human hepatocarcinoma HepG2 cells were maintained in DMEM supplemented with 10% fetal bovine serum.

### Affinity purification of protein complexes

The human cDNAs of AhR (BC070080) and PKM2 (BC000481) were purchased from Open Biosystems (Alabama, USA). Transduction of stable FLAG/HA-tagged AhR or PKM2-expressing HeLa S3 cells and affinity purification of AhR or PKM2 complex from a solubilized chromatin fraction were according to a previous report (12).

### Co-immunoprecipitation

All procedures were performed at 4°C or on ice. For co-immunoprecipitation using the chromatin fraction, the chromatin fraction was incubated with anti-AhR antibody (N-19, Santa Cruz Biotechnology, CA, USA), anti-PDC-E2 antibody (ab110332, Abcam, Cambridge, UK), normal goat IgG (sc-2028, Santa Cruz Biotechnology) or normal mouse IgG (sc-2025, Santa Cruz Biotechnology) for 2 h. After protein G-Agarose (Life Technologies, CA, USA) was added to the sample, the sample was incubated for further 1 h. Immunoprecipitates were pelleted, washed with binding buffer (20 mM Tris–HCl pH 8.0, 10 mM KCl, 0.2 mM, 0.2 mM ethylenediaminetetraacetic acid, 1 mM 2-mercaptoethanol, 0.2 mM phenylmethylsulfonyl fluoride, 10% glycerol, 0.1% Tween 20) three times and then eluted in 100 mM glycine–HCl pH 2.5 by incubation for 5 min. The eluate was neutralized by addition of 1 M Tris–HCl pH 7.3. The eluate was analyzed by western blotting. For co-immunoprecipitation using whole cell lysate, HeLa S3 cells were harvested and lysed in Cell Lysis buffer (150 mM NaCl, 0.5% NP-40, 10% glycerol, 2 mM β-mercaptoethanol, 0.2 mM phenylmethylsulfonyl fluoride, 10 mM β-glycerophosphate) after experimental treatment. Lysates were centrifuged at 15 000 rpm for 30 min and the supernatant was pre-cleared with normal rabbit IgG (#2729, Cell Signaling Technology, MA, USA) and protein G-Agarose (Life Technologies, CA, USA). After centrifugation at 3000 rpm for 2 min, the supernatant was incubated with anti-PKM2 antibody (raised in this study as shown in the ‘Western blotting’ section) or normal rabbit IgG conjugated with Dynabeads Protein G (Life Technologies). Immunoprecipitates were pelleted, washed with Cell Lysis buffer twice, followed by phosphate-buffered saline and then eluted in 100 mM glycine–HCl pH 2.5 by incubation for 1 h on ice. The eluate was neutralized by addition of 1 M Tris–HCl pH 8.0. The eluate was analyzed by western blotting.

### Western blotting

Western blotting was carried out according to standard methods. We used the following antibodies; anti-AhR (H-211), anti-GAPDH (FL-335) and anti-PDC-E2 (C-1) antibodies (Santa Cruz Biotechnology), anti-beta-actin (ab8227) and anti-PDC-E2 (ab110332) antibodies (Abcam), anti-GAPDH antibody (MA1–22670, Thermo, MA, USA), anti-cytochrome c antibody (556433,BD Pharmingen, NJ, USA), anti-histone H3 (#9715), anti-FLAG (#2368) and anti-PKM2 (#4053) antibodies (Cell Signaling Technology). Polyclonal anti-PKM2 antibody against a PKM2-specific peptide (LRRLAPITSDPTEATAVGAV) was raised in rabbits.

### Stable knockdown

Two different 19-nt target sequences were designed: shPKM2#1-F, 5′- GCTGTGGCTCTAGACACTA-3′; shPKM2#1-R, 5′- TAGTGTCTAGAGCCACAGC -3′; shPKM2#2-F, 5′-GTTCGGAGGTTTGATGAAA-3′; shPKM2#2-R, 5′-TTTCATCAAACCTCCGAAC-3′, siAhR#1-F, 5′- CAGACAGTAGTCTGTTATA-3′; shAhR#1-R, 5′- TATAACAGACTACTGTCTG -3′; shAhR#2-F, 5′- CAGCTGAATTAAATAACAT-3′; shAhR#2-R, 5′- ATGTTATTTAATTCAGCTG-3′. The 60-bp oligonucleotides were synthesized according to the instruction manual for pSUPER.retro.puro (Oligo Engine, Seattle, WA, USA). Retroviral production was used to generate HeLa S3 cells stably expressing shRNAs specific for PKM2 or AhR, and stable clones were selected with puromycin.

### Real-time RT-PCR

Total RNA was extracted using an RNeasy Mini kit (Qiagen, Hilden, Germany) and DNA was on-column digested using RNase-free DNase Set (Qiagen) during total RNA extraction. cDNA was synthesized from total RNA using a QuantiTect Reverse Transcription kit (Qiagen). The synthesized cDNA was amplified using a QuantiFast SYBR Green polymerase chain reaction (PCR) kit (QIAGEN). Primer sequences were as follows: *CYP1A1*-F, 5′-CACCATCCCCCACAGCAC-3′; *CYP1A1*-R, 5′-ACAAAGACACAACGCCCCTT-3′; *CYP1B1*-F, 5′- CTGGCACTGACGACGCCAAGAGACT -3′; *CYP1B1*-R, 5′-TGGTCTGCTGGATGGACAGCGGGTT-3′; *CYP1A2*-F, 5′-CCAACGTCATTGGTGCCATG-3′; *CYP1A2*-R, 5′-GTGATGTCCCGGACACTGTTC-3′; *β_2_-microglobulin* (*β2M*)-F, 5′-ACTGAATTCACCCCCACTGA-3′; *β2M*-R, 5′-CCTCCATGATGCTGCTTACA-3′. PCR was performed using a Thermal Cycler Dice Real Time System TP800 (Takara). The mRNA levels were normalized against *β2M* mRNA.

### Overexpression of PKM2

The human cDNA of *PKM2* (BC000481) was cloned into pCMV-Tag2A (Stratagene). HepG2 cells were transiently transfected with pCMV-Tag2A/PKM2 using Lipofectamine 2000 (Invitrogen). About 24 h after transfection, the cells were used for further experiments.

### ChIP

After treatment with 100 nM indirubin for 45 min, HeLa S3 cells were fixed with 1% formaldehyde at room temperature for 10 min (for histone) or 20 min (for the other proteins) and the fixation reaction was stopped with 125 mM glycine. DNA was immunoprecipitated from sonicated cell lysates. Antibodies used were as follows: normal rabbit IgG (Cell Signaling Technology), AhR (N-19) (Santa Cruz Biotechnology), p300 (Abcam), acetyl-histone H3 (K9) (Millipore, MA, USA) and PKM2 (this study). The immunoprecipitated DNA was purified and real-time PCR was performed using the following primers: *CYP1A1* enhancer-F, 5′-CTTCGCCATCCATTCC-3′; *CYP1A1* enhancer-R, 5′-GGGACTCCTCTTCGTC-3′; *CYP1B1* enhancer-F, 5′-GGCAGCGCCCAGGGATATGACTGGA-3′; *CYP1B1* enhancer-R, 5′-CGGAGAGTGGCAGGAGGAGGCGAAT-3′; *CYP1A2* enhancer-F, 5′-GATTCTCACGGCAAGAAGGACTCTC-3′; *CYP1A2* enhancer-R, 5′-CCAGCCAGGTATGTGCGTGTTTGTA-3′; *CYP1A1* coding region-F, 5′- CATGTCGGCCACGGAGTTTCTTC-3′; *CYP1A1* coding region-R, 5′- ACAGTGCCAGGTGCGGGTTCTTTC-3′. As a negative control of genome region, SimpleChIP Human α Satellite Repeat Primers (Cell signaling) was used. Quantitative analysis was confirmed by comparing the dilution series of the input fraction.

### Rescue experiment

The human cDNA of *PKM2* (BC000481) was cloned into p3xFLAG CMV14 (Sigma-Aldrich). Site-directed mutagenesis for shPKM2-resistant PKM2 and PKM2 K367M mutant was performed using a PrimeSTAR Mutagenesis Basal Kit (Takara, Shiga, Japan). HeLa S3 cells expressing shPKM2 #1 were transiently transfected with p3xFLAG CMV14, p3xFLAG CMV14/shPKM2 #1-resistant PKM2 or p3xFLAG CMV14/shPKM2 #1-resistant PKM2 K367M using Lipofectamine 2000. About 24 h after transfection, the cells were subjected to further experiments.

### Pyruvate kinase assay

Pyruvate kinase activity was measured according to Christifk *et* *al*. (13). For the pyruvate kinase assay of whole-cell lysates, 1 μg of total protein was used in a volume of 100 μl. After the mixture was incubated at 37°C for 30 min, the reaction was stopped by adding 100 μl of 100 mM glycine–HCl pH 2.5. The mixture was then filtered with an Amicon Ultra 3K device (nominal molecular weight limit, 3000) (Merk, Darmstadt, Germany) by centrifugation for 15 min at 15 000 rpm, 4°C. The NAD^+^ in the filtrate was measured by HPLC (Waters, MA, USA). The sample was injected and separated on a Shin-pack XR-ODS column (3.0 × 75 mm) (Shimadzu, Kyoto, Japan). Elution was carried out as follows (solvent A, 10 mM ammonium acetate; solvent B, methanol): 0–3 min, isocratic with 0% B; 3–15 min, linear gradient to 4% B; 15–20 min, linear gradient to 80% B; 20–25 min, isocratic with 80% B; 25–25.1 min, linear gradient to 0% B; 25.1–36 min, isocratic with 0% B; flow rate, 0.2 ml/min. For detection of NAD^+^, absorbance at 260 nm was monitored. The amount of NAD^+^ was quantified by calculating the peak area ratio of NAD^+^. For the pyruvate kinase assay of the purified nuclear fraction sample, 3 μg of total protein was used. The incubation time was 10 min.

### Subcellular fractionation

Cells were suspended with the six volumes of hypotonic buffer (10 mM Tris–HCl pH 7.3, 10 mM KCl, 1.5 mM MgCl_2_, 0.2 mM phenylmethylsulfonyl fluoride, 10 mM β-glycerophosphate and 10 mM β-mercaptoethanol) and centrifuged at 1300 x *g* for 5 min. The cell pellet was resuspended in an equal volume of hypotonic buffer. After incubation on ice for 10 min, the cells were homogenized by forcing them through a 25-gauge needle so that at least 80% of cells were broken and nuclei were exposed. The nuclei were pelleted by centrifugation at 600 x *g* for 10 min. The supernatant was centrifuged at 8000 x *g* for 10 min. The pellet was washed with 0.34 M sucrose in Tris, KCl, MgCl_2_ (TKM) buffer (50 mM Tris–HCl pH 7.3, 25 mM KCl, 5 mM MgCl_2_, 0.2 mM phenylmethylsulfonyl fluoride and 10 mM β-glycerophosphate), suspended in 0.34 M sucrose in TKM buffer and retained as a crude mitochondrial fraction. The nuclear pellet was suspended in 0.25 M sucrose in TKM buffer, layered over 1.6 M sucrose in TKM buffer and centrifuged at 13 000 rpm for 30 min. The nuclear pellet was washed with 0.25 M sucrose in TKM buffer and resuspended in 0.34 M sucrose in TKM buffer (nuclear fraction). All samples were frozen in liquid nitrogen and stored at −80°C until use. Repeated freezing and thawing of samples was avoided.

### PDC activity assay

A previous report was used as a reference (14). Ten microgram of total protein was used in the PDC assay in a volume of 100 μl. After incubating the mixture at 37°C for 10 min, the reaction was stopped by adding 100 μl of 100 mM glycine–HCl pH 2.5. The mixture was then filtered with an Amicon Ultra 3K device by centrifugation for 15 min at 15 000 rpm, 4°C. The product, acetyl-CoA, was measured by LC/MS/MS. LC/MS/MS analysis was carried out using a Quattro Ultima Pt triple stage quadrupole mass spectrometer (Waters) equipped with a Waters LC system. The LC separation was according to a previous report (15). Multi-reaction monitoring was performed in negative ion mode using nitrogen as the nebulizing gas. Experimental conditions were as follows: ion source temperature, 130°C; desolvation temperature, 380°C; capillary voltage, 2.4 kV; cone voltage, 35 V; collision energy, 15 eV; desolvation gas flow rate, 700 L/h; cone gas flow rate, 35 L/h; collision gas, argon. The MRM transition for acetyl-CoA was *m/z* 808.0 → 407.7. The amount of acetyl-CoA was quantified by calculating the peak area ratio of acetyl-CoA. The kinetics parameters (*K*_m_ and *V*_max_) were fitted to a Hanes–Woolf plot.

## RESULTS

### PKM2, PDC-E2 and p300 constitute a complex with AhR on chromatin

**Figure 1.**
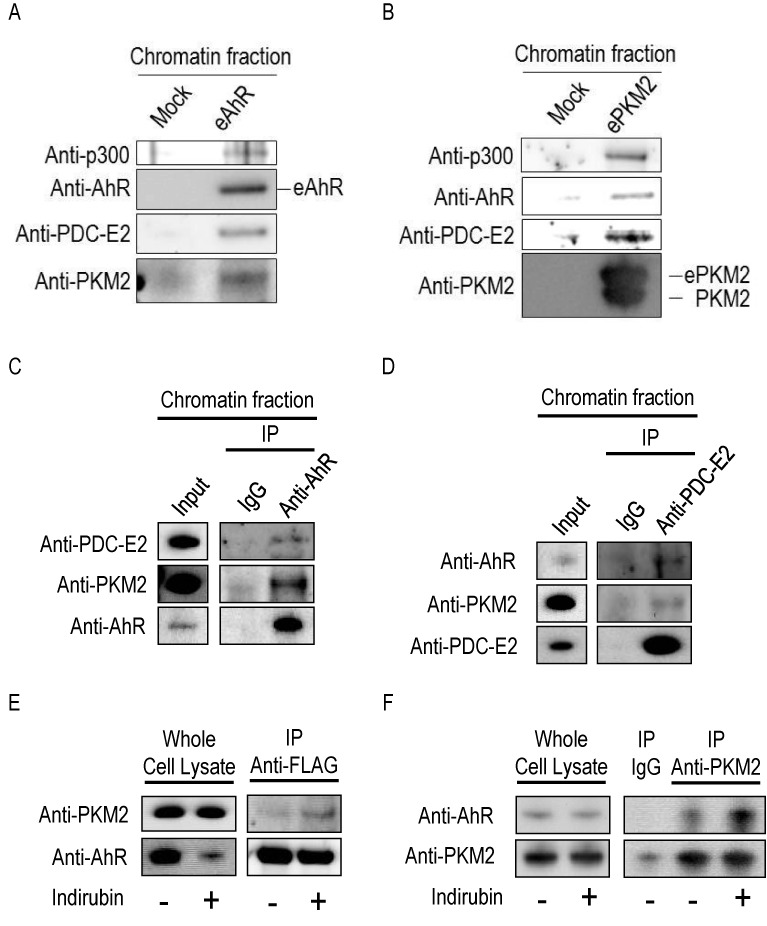
AhR, PKM2, PDC-E2 and p300 constitute a complex in the chromatin fraction. (**A**) eAhR complex was purified from a solubilized chromatin fraction of stably transduced HeLa S3 cells after treatment with 100 nM indirubin for 3 h, and analyzed by western blotting. A mock purification was performed using non-transduced HeLa S3 cells. (**B**) ePKM2 complex was purified from a solubilized chromatin fraction of stably transduced HeLa S3 cells after treatment with 100 nM indirubin for 3 h and analyzed by western blotting. A mock purification was performed using non-transduced HeLa S3 cells. (**C**) Endogenous AhR was immunoprecipitated by anti-AhR antibody from the chromatin fraction of HeLa S3 cells. (**D**) Endogenous PDC-E2 was immunoprecipitated by anti-PDC-E2 antibody from the chromatin fraction of HeLa S3 cells. (**E**) eAhR complex was immunoaffinity-purified by anti-FLAG antibody-conjugated agarose from whole-cell lysates of eAhR-expressing HeLa S3 cells not treated (−) or treated (+) with 100 nM indirubin for 3 h. F. PKM2 complex was immunoprecipitated using anti-PKM2 antibody from whole-cell lysates of HeLa S3 cells not treated (−) or treated (+) with 100 nM indirubin for 1 h.

To identify components of the ligand-stimulated AhR complex, HeLa S3 cells stably expressing FLAG-HA-tagged AhR (eAhR) were treated with 100 nM indirubin, a potent endogenous AhR ligand (16), for 3 h. The chromatin fraction was prepared as indicated in the ‘Experimental procedures’ section and eAhR complex was purified by affinity chromatography on anti-FLAG antibody-conjugated agarose. We detected PKM2, PDC-E2 and p300 in the eAhR complex (Figure 1A). These proteins were not detected in the mock control, indicating specific interactions between AhR and these proteins. To confirm the interactions, FLAG-HA-tagged PKM2 (ePKM2) complex was purified from the chromatin fraction of HeLa S3 cells stably expressing ePKM2 under the same conditions. Consistent with the previous co-immunoprecipitation experiment, we detected AhR, PDC-E2 and p300 in the ePKM2 complex but not in the mock control (Figure 1B). Endogenous PKM2 was also detected in the PKM2 complex since PKM2 can exist as a dimer or tetramer (reviewed in (17)). We also detected PKM2 and PDC-E2 in immuniprecipitate using anti-AhR antibody from the chromatin fraction of HeLa S3 cells (Figure 1C) and AhR and PKM2 in immuniprecipitate using anti-PDC-E2 antibody (Figure 1D). These findings indicate that PKM2, PDC-E2, p300 and AhR constitute a complex on chromatin.

### PKM2 enhances AhR-mediated transcriptional activation

We next focused on the AhR–PKM2 interaction. Co-immunoprecipitation using anti-FLAG antibody or anti-PKM2 antibody confirmed the interaction between AhR and PKM2, and the interaction was enhanced by indirubin treatment (Figure 1E and F).

**Figure 2.**
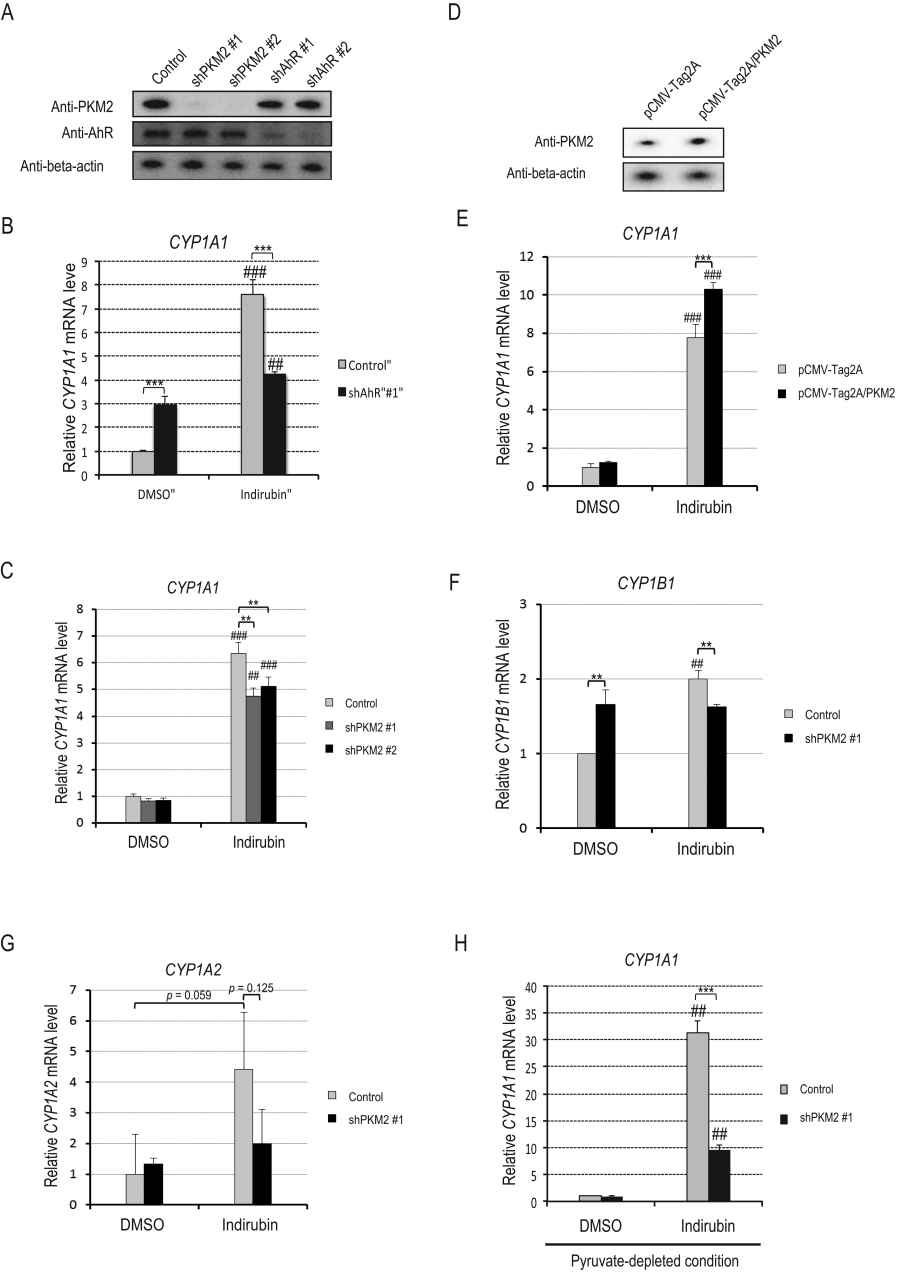
PKM2 promotes AhR transactivation. (**A**) HeLa S3 cells were transduced with two retroviruses encoding different shRNAs targeting PKM2 (shPKM2) or two retroviruses encoding different shRNAs targeting AhR (shAhR). Non-transduced HeLa S3 cells were used as controls. Whole-cell lysates were subjected to western blotting. (**B**) After AhR in HeLa S3 cells was knocked down with shRNA targeting different sites of the *AhR* gene (shAhR #1), the cells were exposed to DMSO or 10 nM indirubin for 2 h. *CYP1A1* mRNA levels were measured by real-time RT-PCR (mean ± SD, *n* = 3). ##*P* < 0.01, ###*P* < 0.001 versus DMSO, ***P < 0.001 (Student's *t*-test). (**C**) After PKM2 in HeLa S3 cells was knocked down with shRNAs targeting different sites of the *PKM2* gene (shPKM2 #1, #2), the cells were exposed to DMSO or 10 nM indirubin for 2 h. *CYP1A1* mRNA levels were measured by real-time RT-PCR (mean ± SD, *n* = 3). ##*P* < 0.01, ###*P* < 0.001 versus DMSO, **P < 0.01 (Student's *t*-test). (**D**) HeLa S3 cells were transfected with pCMV-Tag2A (vector control) or pCMV-Tag2A/PKM2. After 24 h, whole-cell lysates were subjected to western blotting. (**E**) After HeLa S3 cells transfected with pCMV-Tag2A (vector control) or pCMV-Tag2A/PKM2 were exposed to DMSO or 100 nM indirubin for 6 h, *CYP1A1* mRNA levels were measured by real-time RT-PCR (mean ± SD, *n* = 5). ###*P* < 0.001 versus DMSO, ***P < 0.001 (Student's *t*-test). (**F** and **G**) HeLa S3 cells non-transfected (control) or transfected with shPKM2 #1 were exposed to DMSO or 10 nM indirubin for 2 h. mRNA levels of *CYP1B1* (F) and *CYP1A2* (G) were measured by real-time RT-PCR (mean ± SD, *n* = 3). ##*P* < 0.01 versus DMSO, **P < 0.01 (Student's *t*-test). (**H**) HeLa S3 cells were conditioned in medium without pyruvate for 24 h and treated with 10 nM indirubin for 2 h. Then, *CYP1A1* mRNA levels were measured by real-time RT-PCR (mean ± SD, *n* = 3). ##*P* < 0.01 vs. DMSO, ***P < 0.001 (Student's *t*-test).

We investigated the significance of the AhR-PKM2 interaction in AhR-mediated transcriptional activation. Knockdown experiment indicates that neither AhR nor PKM2 affect each other in protein expression level (Figure 2A). As reported, AhR knockdown significantly suppressed indirubin-induced *CYP1A1* mRNA expression (Figure 2B). PKM2 knockdown resulted in a significant reduction in *CYP1A1* mRNA level in cells exposed to indirubin (Figure 2C). Conversely, PKM2 overexpression significantly increased *CYP1A1* mRNA expression in indirubin-treated cells (Figure 2D and E). We tested the other AhR-target genes *CYP1B1* and *CYP1A2*. PKM2 knockdown suppressed *CYP1B1* mRNA induction by indirubin (Figure 2F). PKM2 knockdown also showed tendency to suppress *CYP1A2* mRNA induction by indirubin although the effect was not statistically significant (Figure 2G).

In cancer cells, lactate dehydrogenase, which converts pyruvate to lactate, is overexpressed and the pyruvate concentration is low compared with normal cells (18). The fact prompted us to elucidate a significance of PKM2 in AhR-mediated transcriptional activation in pyruvate-depleted condition. Figure 2H shows that the suppression of indirubin-induced *CYP1A1* mRNA expression by PKM2 knockdown was more drastic in pyruvate-depleted condition than that in normal condition (Figure 2C). These data are consistent with the idea that PKM2 enhances AhR transactivation.

### PKM2 is recruited to gene enhancers of the AhR-target genes in an AhR-dependent manner

**Figure 3.**
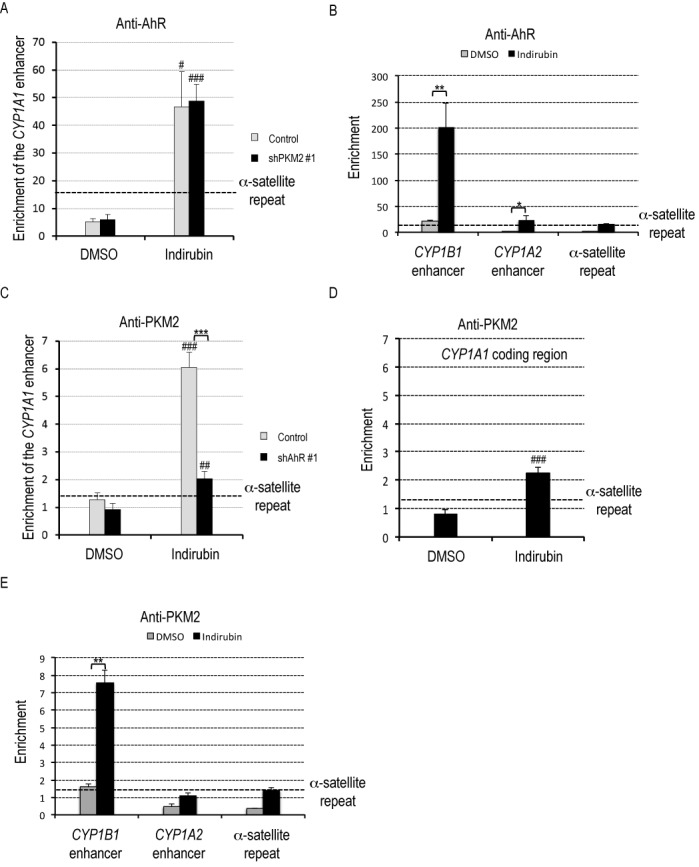
PKM2 is recruited to gene enhancers of the AhR-target genes in an AhR-dependent manner. (**A** and **B**) HeLa S3 cells non-transfected (control) or transfected with shPKM2 #1 were exposed to DMSO or 100 nM indirubin for 45 min. ChIP assays were performed with IgG or anti-AhR antibody (mean ± SD, *n* = 3). α-satellite repeat was used as a negative control of genome region. #*P* < 0.05, ###*P* < 0.001 versus DMSO, *P < 0.05, **P < 0.01 (Student's *t*-test). (**C**, **D** and **E**) HeLa S3 cells non-transfected (control) or transfected with shAhR #1 were exposed to DMSO or 100 nM indirubin for 45 min. ChIP assays were performed with IgG or anti-PKM2 antibody (mean ± SD, *n* = 3). α-satellite repeat was used as a negative control of genome region. ##*P* < 0.01, ###*P* < 0.001 versus DMSO, **P < 0.01 (Student's *t*-test).

Next, to determine whether PKM2 directly regulates AhR-mediated transcription, a ChIP assay was performed. After HeLa S3 cells were exposed to 100 nM indirubin for 45 min, the presence of AhR was significantly increased at the enhancers of *CYP1A1, CYP1B1* and *CYP1A2* (Figure 3A and B). PKM2 depletion did not affect AhR occupancy at the *CYP1A1* enhancer (Figure 3A), indicating that recruitment of AhR to the gene enhancers was independent of PKM2. Interestingly, PKM2 was significantly enriched at the *CYP1A1* enhancer by indirubin treatment (Figure 3C). PKM2 was also enriched at the coding region of *CYP1A1* by indirubin treatment but the increase was less than PKM2 at the *CYP1A1* promoter (Figure 3D). This data suggests that PKM2 preferably binds to the enhancer rather than the gene body. PKM2 presence was significantly decreased in AhR-depleted cells (Figure 3C), indicating that PKM2 is recruited to the enhancers in an AhR-dependent manner. We also tested the enhancers of *CYP1B1* and *CYP1A2*. PKM2 was significantly enriched at the *CYP1B1* enhancer by indirubin treatment. In case of the *CYP1A2* enhancer, the significant enrichment of PKM2 was not observed (Figure 3E). These data suggest that PKM2 directly controls transactivation of at least a range of AhR-target genes.

### The pyruvate kinase activity of PKM2 enhances acetylation of K9 in histone H3 at the CYP1A1 gene enhancer

**Figure 4.**
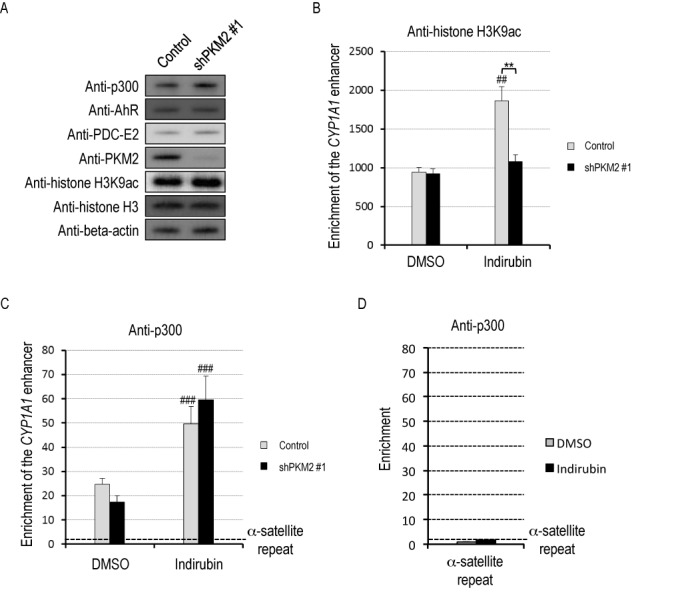
PKM2 promotes histone acetylation at the *CYP1A1* enhancer. (**A**) Whole-cell lysates of HeLa S3 cells non-transfected (control) or transfected with shPKM2 #1 were subjected to western blotting. (**B**) HeLa S3 cells non-transfected (control) or transfected with shPKM2#1 were exposed to DMSO or 100 nM indirubin for 45 min. ChIP assays were performed with IgG or anti-histone H3K9ac antibody (mean ± SD, *n* = 3). ##*P* < 0.01 versus DMSO, **P < 0.01 (Student's *t*-test). (**C** and **D**) HeLa S3 cells non-transfected (control) or transfected with shPKM2 #1 were exposed to DMSO or 100 nM indirubin for 45 min. ChIP assays were performed with IgG or anti-p300 (mean ± SD, *n* = 3). α-satellite repeat was used as a negative control of genome region. ###*P* < 0.001 versus DMSO (Student's *t*-test).

To elucidate a role for PKM2 at the gene enhancer, we next examined whether PKM2 affects histone acetylation, especially acetylation at lysine 9 of histone H3 (H3K9), which is known to be associated with activation of transcription. PKM2 knockdown did not apparently affect overall H3K9 acetylation levels or total amount of proteins identified from AhR/PKM2 complex, compared with normal HeLa S3 cells (Figure 4A). However, ChIP assays revealed that PKM2 knockdown significantly decreased H3K9 acetylation levels at the *CYP1A1* enhancer in cells treated with indirubin (Figure 4B). Since p300 is a known transcriptional co-activator for AhR transactivation (19,20), we next investigated whether the observed reduction in histone acetylation could be attributed to reduced p300 recruitment at the *CYP1A1* enhancer. PKM2 knockdown in cells did not affect recruitment of p300 to the *CYP1A1* enhancer (Figure 4C and D).

**Figure 5.**
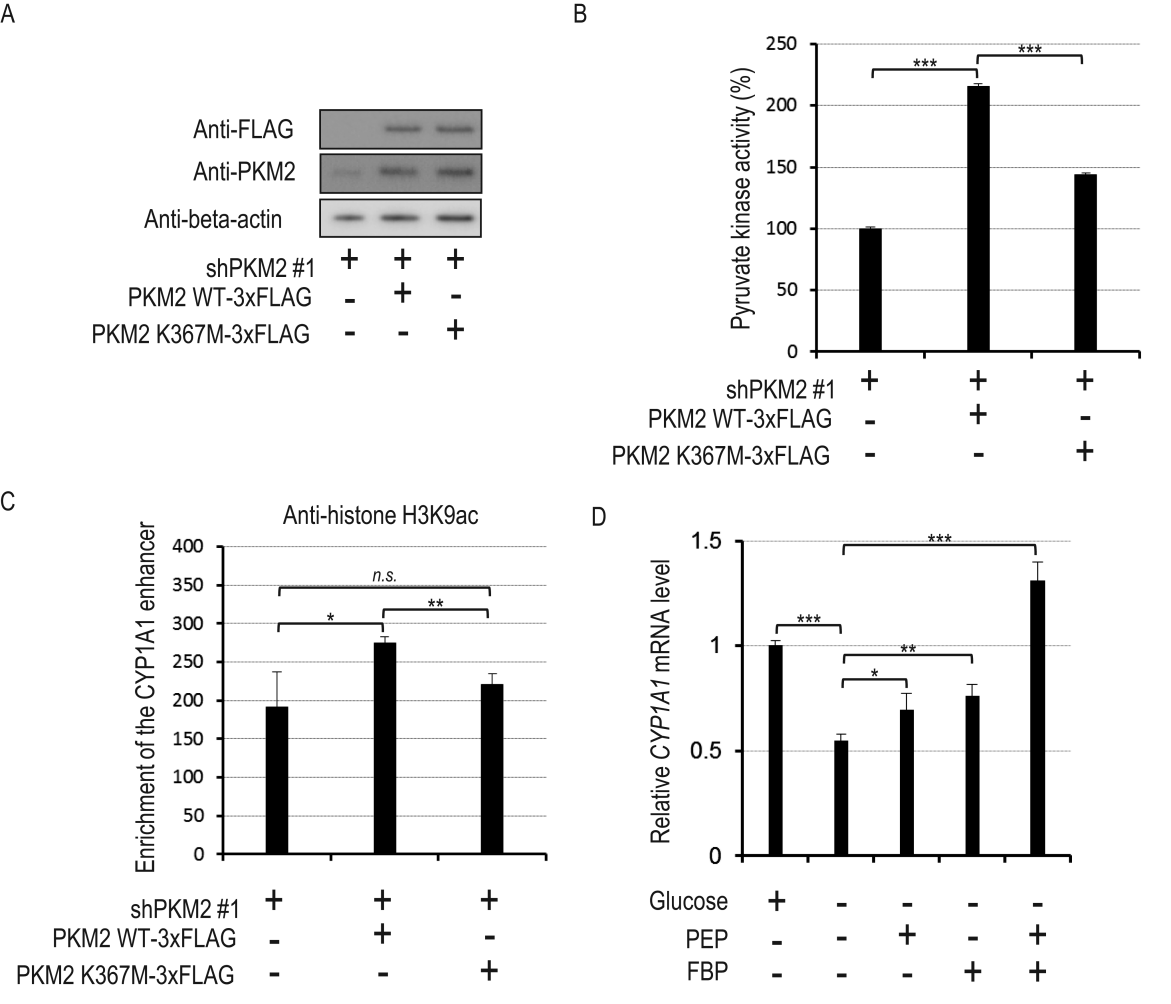
Pyruvate kinase activity of PKM2 promotes histone acetylation at the *CYP1A1* enhancer. (**A**) HeLa S3 cells expressing shPKM2 #1 were transfected with empty vector, vector encoding shPKM2 #1-resistant PKM2 wild-type (WT)-3xFLAG (wild-type PKM2) or PKM2 K367M-3xFLAG (mutant type PKM2). Whole-cell lysates were subjected to western blotting. (**B**) HeLa S3 cells expressing shPKM2 #1 were transfected with empty vector, vector encoding shPKM2 #1-resistant PKM2 WT-3xFLAG or PKM2 K367M-3xFLAG. After cell lysis, pyruvate kinase activity was assayed (mean ± SD, *n* = 3). ***P < 0.001 (Student's *t*-test). (**C**) HeLa S3 cells expressing shPKM2 #1 were transfected with empty vector, vector encoding shPKM2 #1-resistant PKM2 WT-3xFLAG or shPKM2 #1-resistant PKM2 K367M-3xFLAG and exposed to 100 nM indiruin for 45 min. ChIP assays were performed with IgG or anti-histone H3K9ac antibody (mean ± SD, *n* = 3). n.s., not significant; *P < 0.05, **P < 0.01 (Student's *t*-test). (**D**) HeLa S3 cells were conditioned in medium with 4.5 g/l glucose or without glucose for 24 h and treated with 100 nM indirubin or co-treated with 100 nM indirubin and 5 mM PEP and/or 5 mM FBP for 2 h. Then, *CYP1A1* mRNA levels were measured by real-time RT-PCR (mean ± SD, *n* = 3). *P < 0.05, **P < 0.01, ***P < 0.001 (Student's *t*-test).

To determine whether histone acetylation at the *CYP1A1* enhancer requires the enzymatic activity of PKM2, we performed rescue experiments. FLAG- tagged, shRNA-resistant wild-type PKM2 or a PKM2 K367M mutant, which lacks pyruvate kinase activity (21), was expressed in PKM2-knockdown HeLa S3 cells (Figure 5A and B), the cells were treated with 100 nM indirubin for 45 min, and then ChIP assays were performed. As shown in Figure 5C, overexpression of wild-type PKM2 increased H3K9 acetylation at the *CYP1A1* enhancer, whereas overexpression of the PKM2 K367M mutant failed to do so, indicating that efficient histone acetylation at the *CYP1A1* enhancer requires the pyruvate kinase activity of PKM2. Next, we tested whether pyruvate kinase activity affects the transcriptional activity of AhR. Note that PEP is a substrate of PKM2 and a source of pyruvate, and that PKM2 is allosterically activated by a glycolytic metabolite, fructose 1,6-bisphosphate (FBP), which is not the case for PKM1 (22). Therefore, we examined the effects of these two substances (PEP and FBP) on AhR-mediated transactivation. Since PEP and FBP are mainly derived from glucose via glycolysis, in order to potentiate their effects, HeLa S3 cells were conditioned in medium without glucose for 24 h to deplete intracellular PEP and FBP. After glucose depletion, the cells were co-treated with 10 nM indirubin together with 5 mM PEP and/or 5 mM FBP. The *CYP1A1* mRNA level was significantly decreased by glucose depletion, and was moderately rescued by PEP or FBP treatment. On the other hand, co-treatment with PEP and FBP resulted in a synergistic increase in the *CYP1A1* mRNA level (Figure 5D). This result is consistent with an idea that pyruvate kinase activity promotes the transcriptional activity of AhR.

### The nucleus has PDC activity and PDC-E2 is recruited to the CYP1A1 gene enhancer in an AhR ligand-dependent manner

**Figure 6.**
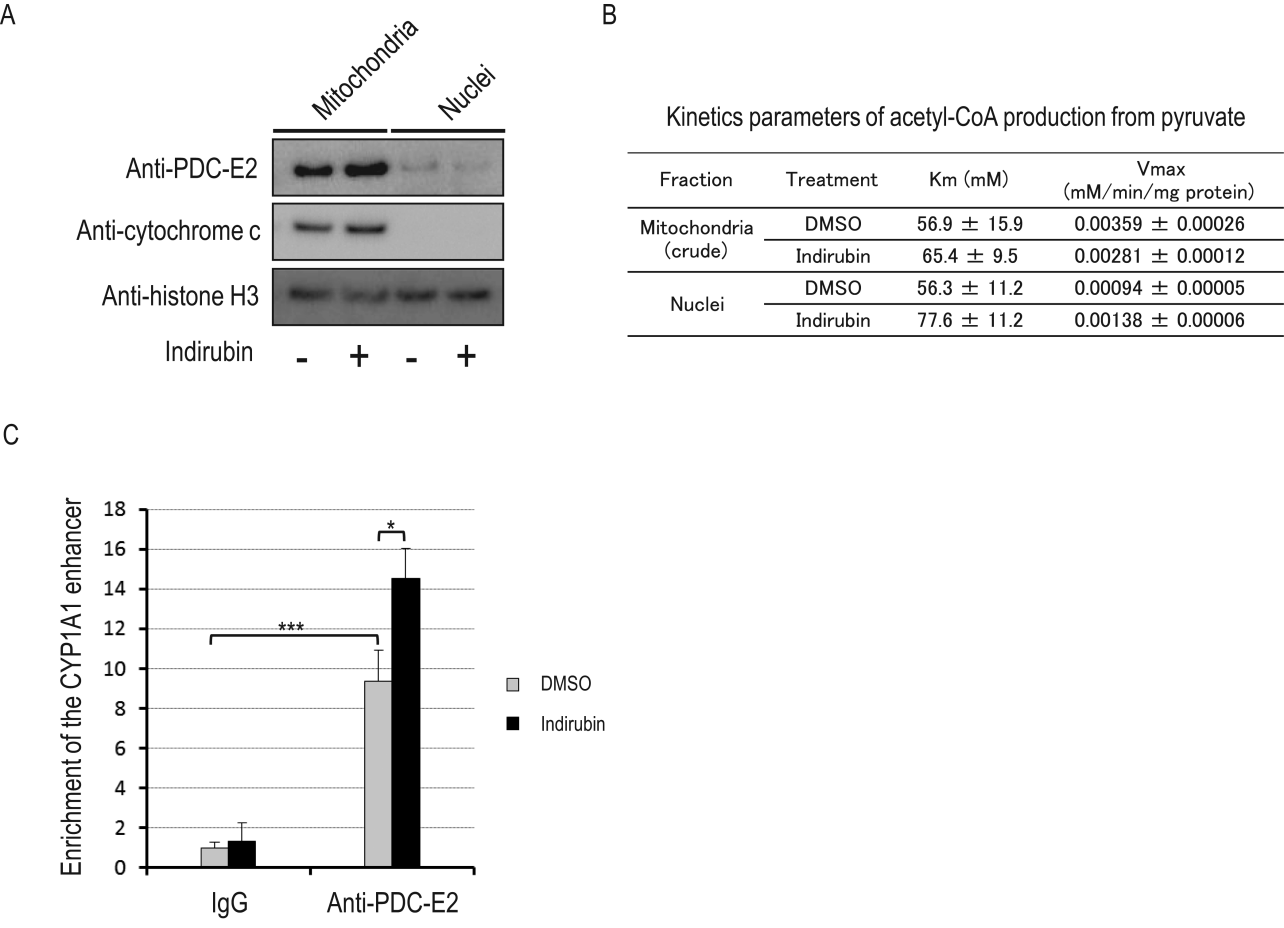
The nucleus has PDC activity and PDC-E2 is recruited to the *CYP1A1* enhancer in an AhR-ligand-dependent manner. (**A**) HeLa S3 cells were fractionated into cytoplasmic, mitochondrial and nuclear fractions. The nuclear fraction was further purified by sucrose density-gradient centrifugation as described in the Experimental procedures section. Lysates of each fraction were subjected to western blotting. (**B**) Kinetics parameters of acetyl-CoA production from pyruvate in each fraction. Each fraction was subjected to a PDC assay as described in the ‘Materials and Methods’ section, and the apparent *K*_m_ for pyruvate and apparent *V*_max_ were determined (mean ± SE, *n* = 3). (**C**) HeLa S3 cells were exposed to 100 nM indirubin for 45 min. ChIP assays were performed with IgG or anti-PDC-E2 antibody (mean ± SD, *n* = 3). *P < 0.05, ***P < 0.001 (Student's *t*-test).

The next question we addressed was why pyruvate kinase activity is necessary for histone acetylation at the *CYP1A1* enhancer. It is well known that pyruvate is used by PDC to generate acetyl-CoA in mitochondria. In this study, we detected PDC-E2 in complexes with AhR and PKM2 immunoprecipitated from nuclear chromatin (Figure 1). Consistent with those findings, we detected PDC-E2 by western blotting in a carefully purified nuclear fraction (Figure 6A). Histone H3 present in the mitochondrial fraction is consistent with previous reports (23,24). Furthermore, the nuclear fraction was able to produce acetyl-CoA from pyruvate (Figure 6B). The *K*_m_ for pyruvate was similar between the mitochondrial and nuclear fractions, but the *V*_max_ of the nuclear fraction was about 16% of that of the mitochondrial fraction, which is fairly consistent with the difference in PDC-E2 protein levels between the two fractions. These results indicate that PDC in the nucleus is equally able to utilize pyruvate to make acetyl-CoA as PDC in the mitochondria, consistent with a recent report (4). This prompted us to ask whether PDC-E2 is recruited to the *CYP1A1* enhancer. After HeLa S3 cells were treated with 100 nM indirubin for 45 min, binding of PDC-E2 to the *CYP1A1* enhancer was determined by ChIP assay. Enrichment of PDC-E2 was significantly increased by indirubin treatment (Figure 6C), indicating that PDC-E2 is recruited to the *CYP1A1* enhancer following ligand activation of AhR.

## DISCUSSION

**Figure 7.**
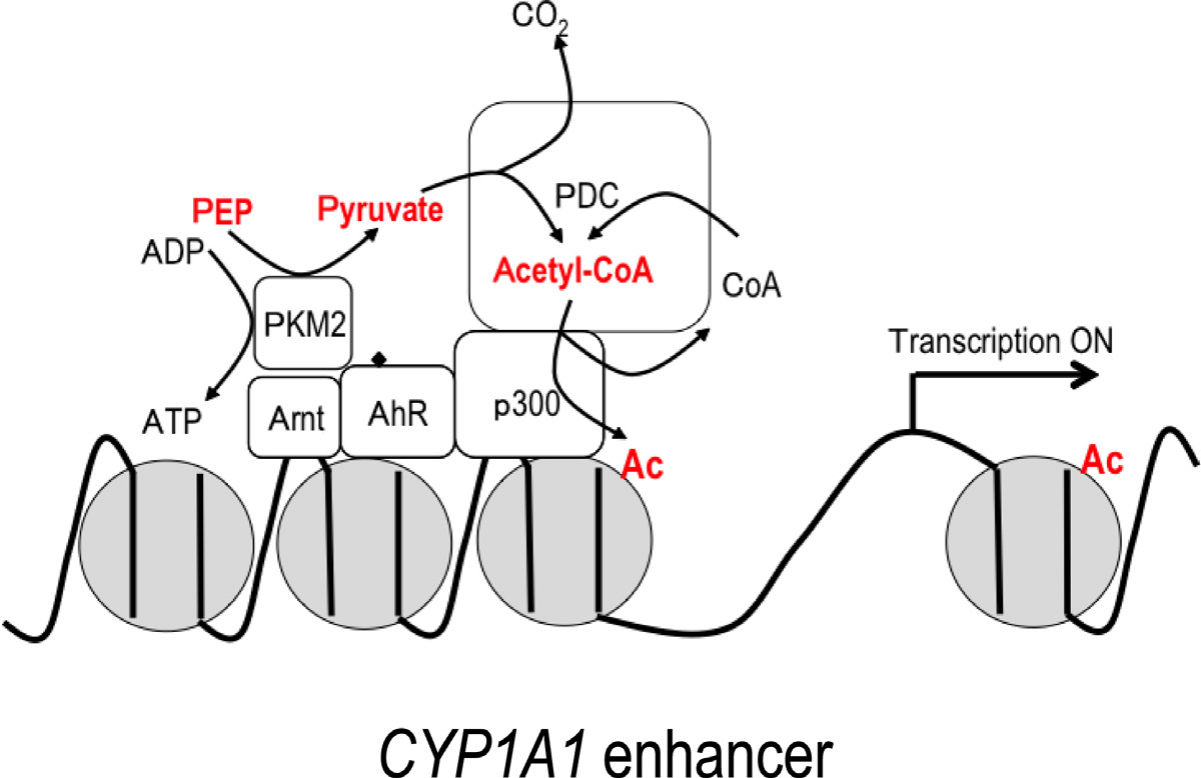
Hypothetical model based on this study. Nuclear PKM2 and PDC coordinately serve as a ‘supplier’ of acetyl-CoA in the *CYP1A1* enhancer region. The CoA byproduct of histone acetylation is immediately recycled to acetyl-CoA by PDC.

We showed here that p300, PDC-E2 and PKM2 are recruited to the *CYP1A1* gene enhancer together with AhR, and these factors constitute a complex on chromatin. We also found that PDC activity, which produces acetyl-CoA from pyruvate and CoA, and pyruvate kinase activity, which produces pyruvate and ATP from PEP and ADP, are present in the nucleus (data not shown). On the other hand, the fact that PKM2 knockdown did not affect bulk histone acetylation (Figure 4A) suggests that PKM2 might control local histone acetylation rather than bulk acetylation. Together, we suggest that these enzymes are assembled to construct a local acetyl-CoA supply system for histone acetylation at the *CYP1A1* gene enhancer (Figure 7). In this model, CoA could be immediately recycled to acetyl-CoA by PDC using pyruvate. Thus, by this mechanism, the acetyl-CoA concentration in proximity to p300 at the gene enhancer could be stably maintained at a high level when and where it is required.

Among endogenous AhR ligands reported (16,25,26), the potent AhR ligand indirubin also functions as an anti-cancer drug to inhibit cyclin-dependent kinases (27). Thus, accumulation of the highly-insoluble compound is harmful especially to proliferating cells such as cancer and fetal cells. On the other hand, CYP1A1 induced by AhR activation can easily metabolize indirubin (28), emphasizing the importance of the AhR-mediated detoxification system in proliferating cells. Previous reports show that PKM2 in tumor and fetal cells is expressed at much higher levels than in the adult (29–32), whereas AhR is expressed in tumor, fetal and adult tissues. Our results indicate that PKM2 would confer an advantage to proliferating cells by allowing them to effectively eliminate harmful endogenous metabolites or exogenous chemicals and thereby support cell proliferation.

In contrast to its role in detoxification, AhR also mediates the toxicity of dioxins, which are potent exogenous and highly stable AhR ligands (33–35). Fetuses, especially, are highly-sensitive to dioxins by still unknown mechanisms. Exposure of dioxins to pregnant mice can induce fetal malformations such as hydronephrosis and cleft palate without severe toxicity to the dams, and these teratogenic effects are dependent on AhR expression (36,37). Enhancement of AhR activation by PKM2 is thought to occur preferentially in the fetus, resulting in fetal hypersensitivity to dioxins. On the other hand, it should be noted that the Ah-responsiveness of different AhR ligands is structure-, cell context- and response-dependent (38).

Our model is also interesting in terms of the significance of PKM2 in tumorigenesis. Previous studies have shown that PKM2 is a major component of the Warburg effect and regulates switching of glucose utilization between energy production and synthesis of cellular building blocks in proliferating cells (13,17). In cancer cells, lactate dehydrogenase, which converts pyruvate to lactate, is overexpressed and the pyruvate concentration is low compared with normal cells (18). Even in this situation, as envisioned by our model, cancer cells could still supply acetyl-CoA to sites of transcription by using nuclear PKM2 to generate pyruvate from its abundant substrate, PEP.

## References

[1] Zentner G.E., Henikoff S. (2013). Regulation of nucleosome dynamics by histone modifications. Nat. Struct. Mol. Biol..

[2] Wellen K.E., Hatzivassiliou G., Sachdeva U.M., Bui T.V., Cross J.R., Thompson C.B. (2009). ATP-citrate lyase links cellular metabolism to histone acetylation. Science.

[3] Takahashi H., McCaffery J.M., Irizarry R.A., Boeke J.D. (2006). Nucleocytosolic acetyl-coenzyme a synthetase is required for histone acetylation and global transcription. Mol. Cell.

[4] Sutendra G., Kinnaird A., Dromparis P., Paulin R., Stenson T.H., Haromy A., Hashimoto K., Zhang N., Flaim E., Michelakis E.D. (2014). A nuclear pyruvate dehydrogenase complex is important for the generation of Acetyl-CoA and histone acetylation. Cell.

[5] Gao X., Wang H., Yang J.J., Liu X., Liu Z.R. (2012). Pyruvate kinase M2 regulates gene transcription by acting as a protein kinase. Mol. Cell.

[6] Lee J., Kim H.K., Han Y.M., Kim J. (2008). Pyruvate kinase isozyme type M2 (PKM2) interacts and cooperates with Oct-4 in regulating transcription. Int. J. Biochem. Cell Biol..

[7] Luo W., Hu H., Chang R., Zhong J., Knabel M., O'Meally R., Cole R.N., Pandey A., Semenza G.L. (2011). Pyruvate kinase M2 is a PHD3-stimulated coactivator for hypoxia-inducible factor 1. Cell.

[8] Yang W., Xia Y., Ji H., Zheng Y., Liang J., Huang W., Gao X., Aldape K., Lu Z. (2011). Nuclear PKM2 regulates beta-catenin transactivation upon EGFR activation. Nature.

[9] Yang W., Xia Y., Hawke D., Li X., Liang J., Xing D., Aldape K., Hunter T., Yung W.K., Lu Z. (2012). PKM2 phosphorylates histone H3 and promotes gene transcription and tumorigenesis. Cell.

[10] Chueh F.Y., Leong K.F., Cronk R.J., Venkitachalam S., Pabich S., Yu C.L. (2011). Nuclear localization of pyruvate dehydrogenase complex-E2 (PDC-E2), a mitochondrial enzyme, and its role in signal transducer and activator of transcription 5 (STAT5)-dependent gene transcription. Cell Signal..

[11] Nebert D.W., Roe A.L., Dieter M.Z., Solis W.A., Yang Y., Dalton T.P. (2000). Role of the aromatic hydrocarbon receptor and [Ah] gene battery in the oxidative stress response, cell cycle control, and apoptosis. Biochem. Pharmacol..

[12] Yusufzai T.M., Tagami H., Nakatani Y., Felsenfeld G. (2004). CTCF tethers an insulator to subnuclear sites, suggesting shared insulator mechanisms across species. Mol. Cell.

[13] Christofk H.R., Vander Heiden M.G., Harris M.H., Ramanathan A., Gerszten R.E., Wei R., Fleming M.D., Schreiber S.L., Cantley L.C. (2008). The M2 splice isoform of pyruvate kinase is important for cancer metabolism and tumour growth. Nature.

[14] Hinman L.M., Blass J.P. (1981). An NADH-linked spectrophotometric assay for pyruvate dehydrogenase complex in crude tissue homogenates. J. Biol. Chem..

[15] Nakamura T., Pluskal T., Nakaseko Y., Yanagida M. (2012). Impaired coenzyme A synthesis in fission yeast causes defective mitosis, quiescence-exit failure, histone hypoacetylation and fragile DNA. Open Biol..

[16] Adachi J., Mori Y., Matsui S., Takigami H., Fujino J., Kitagawa H., Miller C.A. 3rd, Kato T., Saeki K., Matsuda T. (2001). Indirubin and indigo are potent aryl hydrocarbon receptor ligands present in human urine. J. Biol. Chem..

[17] Mazurek S., Boschek C.B., Hugo F., Eigenbrodt E. (2005). Pyruvate kinase type M2 and its role in tumor growth and spreading. Semin. Cancer Biol..

[18] Thangaraju M., Carswell K.N., Prasad P.D., Ganapathy V. (2009). Colon cancer cells maintain low levels of pyruvate to avoid cell death caused by inhibition of HDAC1/HDAC3. Biochem. J..

[19] Garrison P.M., Rogers J.M., Brackney W.R., Denison M.S. (2000). Effects of histone deacetylase inhibitors on the Ah receptor gene promoter. Arch. Biochem. Biophys..

[20] Kobayashi A., Numayama-Tsuruta K., Sogawa K., Fujii-Kuriyama Y. (1997). CBP/p300 functions as a possible transcriptional coactivator of Ah receptor nuclear translocator (Arnt). J. Biochem..

[21] Le Mellay V., Houben R., Troppmair J., Hagemann C., Mazurek S., Frey U., Beigel J., Weber C., Benz R., Eigenbrodt E. (2002). Regulation of glycolysis by Raf protein serine/threonine kinases. Adv. Enzyme Regul..

[22] Noguchi T., Inoue H., Tanaka T. (1986). The M1- and M2-type isozymes of rat pyruvate kinase are produced from the same gene by alternative RNA splicing. J. Biol. Chem..

[23] Choi Y.S., Hoon Jeong J., Min H.K., Jung H.J., Hwang D., Lee S.W., Kim Pak Y. (2011). Shot-gun proteomic analysis of mitochondrial D-loop DNA binding proteins: identification of mitochondrial histones. Mol. Biosyst..

[24] Zanin M.K., Donohue J.M., Everitt B.A. (2010). Evidence that core histone H3 is targeted to the mitochondria in Brassica oleracea. Cell Biol. Int..

[25] Bjeldanes L.F., Kim J.Y., Grose K.R., Bartholomew J.C., Bradfield C.A. (1991). Aromatic hydrocarbon responsiveness-receptor agonists generated from indole-3-carbinol in vitro and in vivo: comparisons with 2,3,7,8-tetrachlorodibenzo-p-dioxin. Proc. Natl. Acad. Sci. U.S.A..

[26] Rannug U., Rannug A., Sjoberg U., Li H., Westerholm R., Bergman J. (1995). Structure elucidation of two tryptophan-derived, high affinity Ah receptor ligands. Chem. Biol..

[27] Hoessel R., Leclerc S., Endicott J.A., Nobel M.E., Lawrie A., Tunnah P., Leost M., Damiens E., Marie D., Marko D. (1999). Indirubin, the active constituent of a Chinese antileukaemia medicine, inhibits cyclin-dependent kinases. Nat. Cell Biol..

[28] Adachi J., Mori Y., Matsui S., Matsuda T. (2004). Comparison of gene expression patterns between 2,3,7,8-tetrachlorodibenzo-p-dioxin and a natural arylhydrocarbon receptor ligand, indirubin. Toxicol. Sci..

[29] Hacker H.J., Steinberg P., Bannasch P. (1998). Pyruvate kinase isoenzyme shift from L-type to M2-type is a late event in hepatocarcinogenesis induced in rats by a choline-deficient/DL-ethionine-supplemented diet. Carcinogenesis.

[30] Harada Y., Nakamura M., Asano A. (1995). Temporally distinctive changes of alternative splicing patterns during myogenic differentiation of C2C12 cells. J. Biochem..

[31] Izumi S., Manabe A., Tomoyasu A., Kihara-Negishi F., Ariga H. (1995). Molecular cloning of the complementary DNA for the mouse pyruvate kinase M-2 gene whose expression is dependent upon cell differentiation. Biochim. Biophys. Acta.

[32] Scott R.J., English V., Noguchi T., Tanaka T., Yeoh G.C. (1988). Pyruvate kinase isoenzyme transitions in cultures of fetal rat hepatocytes. Cell Differ. Dev..

[33] Poland A., Knutson J.C. (1982). 2,3,7,8-tetrachlorodibenzo-p-dioxin and related halogenated aromatic hydrocarbons: examination of the mechanism of toxicity. Annu. Rev. Pharmacol. Toxicol..

[34] Safe S. (1990). Polychlorinated biphenyls (PCBs), dibenzo-p-dioxins (PCDDs), dibenzofurans (PCDFs), and related compounds: environmental and mechanistic considerations which support the development of toxic equivalency factors (TEFs). Crit. Rev. Toxicol..

[35] Couture L.A., Abbott B.D., Birnbaum L.S. (1990). A critical review of the developmental toxicity and teratogenicity of 2,3,7,8-tetrachlorodibenzo-p-dioxin: recent advances toward understanding the mechanism. Teratology.

[36] Mimura J., Yamashita K., Nakamura K., Morita M., Takagi T.N., Nakao K., Ema M., Sogawa K., Yasuda M., Katsuki M. (1997). Loss of teratogenic response to 2,3,7,8-tetrachlorodibenzo-p-dioxin (TCDD) in mice lacking the Ah (dioxin) receptor. Genes Cells.

[37] Peters J.M., Narotsky M.G., Elizondo G., Fernandez-Salguero P.M., Gonzalez F.J., Abbott B.D. (1999). Amelioration of TCDD-induced teratogenesis in aryl hydrocarbon receptor (AhR)-null mice. Toxicol. Sci..

[38] Jin U.H., Lee S.O., Safe S. (2012). Aryl hydrocarbon receptor (AHR)-active pharmaceuticals are selective AHR modulators in MDA-MB-468 and BT474 breast cancer cells. J. Pharmacol. Exp. Ther..

